# Variations in the Levels of Mulberroside A, Oxyresveratrol, and Resveratrol in Mulberries in Different Seasons and during Growth

**DOI:** 10.1155/2013/380692

**Published:** 2013-08-19

**Authors:** Jin Zhou, Shun-xiang Li, Wei Wang, Xiao-yi Guo, Xiang-yang Lu, Xin-pei Yan, Dan Huang, Bao-yang Wei, Liang Cao

**Affiliations:** ^1^School of Pharmacy, Hunan University of Chinese Medicine, Changsha 410208, China; ^2^School of Bioscience & Biotechnology, Hunan Agricultural University, Changsha 410128, China; ^3^Hunan Institute of Sericulture, Changsha 410127, China

## Abstract

This study aimed to investigate the composition of three major stilbenes (mulberroside A, oxyresveratrol, and resveratrol) in different portions of mulberries collected in different seasons and their change molds during growth by high-performance liquid chromatography. Mulberroside A levels were the highest in the bark and roots of *Morus atropurpurea* Roxb, *Morus alba* Linn, and *Morus latifolia* Poir. Oxyresveratrol levels were the highest in roots and stem. Both of these high levels were in September. The amount of resveratrol was very low in all samples. In the stem, *Morus latifolia* Poir contained more mulberroside A than the other two mulberries. Mulberroside A was not detected in the leaves of the three mulberries. In *Morus atropurpurea* Roxb seedlings, the root tended to contain more of the three stilbenes than leaves. The temporal peaks of resveratrol were always ahead of those for oxyresveratrol. The levels of the stilbenes varied in different portions of the varieties of mulberries collected in different season and in the seedlings of *Morus atropurpurea* Roxb.

## 1. Introduction

**Figure 1:**
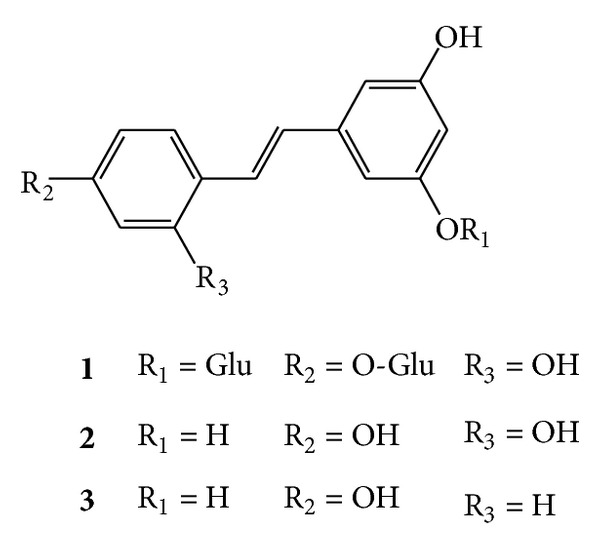
Structures of compounds **1**–**3** identified from mulberry. **1** mulberroside A; **2** oxyresveratrol; **3** resveratrol.

Stilbenic compounds (mulberroside A, oxyresveratrol, and resveratrol) (Figure 1) have been investigated for a wide range of bioactivities including antitumor properties [1], antityrosinase [2], antiviral [3], neuroprotective [4], antioxidant activities [5], and a higher protective effect on DNA [6]. Almost all products are extracted from mulberry cortex for there is no effective method to synthesize these compounds [7]. In addition, little is known about the composition and change patterns of stilbenes during their growth in mulberries. Therefore, this study aimed to investigate the composition of the three major stilbenes in different portions of mulberries collected in different seasons, as well as their change molds during growth, by high-performance liquid chromatography (HPLC).

## 2. Experimental

### 2.1. Chemicals and Materials

Acetonitrile was of HPLC grade (Tedia, USA). Methanol and alcohol were of analytical grade from Tianjin Ke-Miou Reagent Company (Tianjin, China). Ultrapure water was purified by Milli-Q system (Millipore, Bedford, MA, USA). Formic acid was of analytical grade from Tianjin Fuyu Reagent Company (Tianjin, China). Mulberroside A (123-100311), oxyresveratrol (2009031102), and resveratrol (111535-200502) were from Tianjin Kuiqing Reagent Company (Tianjin, China), Mingyuan Reagent Company (Tianjin, China), and Meidi Reagent Company (Zhejiang, China) and were all proved to be above 98% by HPLC analysis. All samples of mulberries were gathered from the Hunan Institute of Sericulture (Changsha, China) and authenticated by Professor Yan XP of the Hunan Institute of Sericulture (Changsha, China).

### 2.2. Apparatus and Chromatographic Conditions

An Agilent 1100 liquid chromatography system (Agilent Technologies Deutschland, Waldbronn, Germany), armed with a quaternary solvent delivery system and ultraviolet detector, was used. All analyses were performed with a Hypersil BDS C18 column (200 mm × 4.6 mm, 5 *μ*m) at a temperature of 40°C. Ultraviolet absorption was set at 320 nm, and an HS-3120 ultrasonic purger was obtained from Jiangsu Hanbon Science & Technology Company (Jiangsu, China). Eluent A (acetonitrile) and B (1.0% aqueous formic acid,* v/v*) were used with the gradient program set as follows: 0–25 min, linear change from A-B (5 : 95, *v/v*) to A-B (30 : 70, *v/v*). Reequilibration interval was 15 min between individual runs with the flow rate 1.0 mL min^−1^. The aliquots of 10 *μ*L were injected each time.

### 2.3. Standard Solutions Preparation

Reference compounds **1**–**3** (**1**—mulberroside A, **2**—oxyresveratrol, **3**—resveratrol) were prepared as follows: following accurately weighed and dissolved in 60% methanol, the compounds were diluted to the concentration ranges of **1** (0.64–404.40 *μ*g mL^−1^), **2** (0.71–444.00 *μ*g mL^−1^), and **3** (0.72–448.00 *μ*g mL^−1^) (see Table 1).

### 2.4. Sample Preparation

Randomly selected samples of mulberry were first air-dried, milled into powder, dried at room temperature until constant weight, and then passed through a 40-mesh sieve, followed by ultrasonic extraction with 25 mL (for 0.5 g) of 60% methanol for 40 min. After that, the solvent was again added to the resultant mixture to make it equal to the original weight prior to the ultrasonic extraction, followed by filtering the supernatant through a 0.45 *μ*m membrane just before HPLC injection. All samples were prepared in triplicate.

Methods to optimize the extraction conditions, calibration graphs, limit of detection (LOD) and quantification (LOQ), and method of validation and application were adapted from the previously reported systems [8, 9]. Briefly, the methods were validated as follows.

### 2.5. Optimization of Extraction Conditions

In the preliminary study, we found that, compared with other methods, ultrasonic extraction was more effective with less interference. Different concentrations of methanol and ethanol were tested for their efficiency as a solvent. As 60% of methanol as a solvent produces the highest yields for all constituents, it was chosen in the current study. The impact of the length of the extraction time on the efficiency of extraction was evaluated as well. Tested with 60% methanol for 10, 20, 40, and 60 mins, respectively, powdered samples extracted the highest amount of constituents when treated for 40 mins. When column temperature was maintained at 40°C instead of 20 or 30°C, optimized separation was achieved. Various mobile phase compositions were also tested. Results show that water/acetonitrile mixture, not methanol/water mixture, can obtain satisfactory resolution. Addition of acid (0.5% formic acid, 0.5% acetic acid, and 1.0% formic acid) in the mobile phase improves resolution and reduces the peak tailing of the target compounds, with the best results obtained when using acetonitrile/water mixture with 1.0% formic acid. The previously mentioned optimized conditions were used in the current study. According to the absorption maxima of three standards on the ultraviolet spectrum, with three-dimensional chromatograms of HPLC-DAD detection, the wavelength of 320 nm was used in the study.

### 2.6. Calibration Graphs, LODs, and LOQs

The concentration of the compounds was determined by external standard method. Linear regression analyses for each compound were conducted by plotting the peak area versus concentration. The calibration curve for each compound was composed of six points representing six different concentrations in triplicate. The results are shown in Table 1. All the compounds show linearity (*r* ^2^ > 0.9999) in a relatively wide concentration range.

The LOD and LOQ for each compound under the chromatographic conditions were obtained by measuring the amount of analytical background (Table 1). The signal-to-noise (S/N) ratio for each compound obtained by injecting a series of solutions is **3** for LOD and 10 for LOQ.

### 2.7. Method Validation

The repeatability of the method was tested by intra- and interday variability. Six replicate samples were extracted and analyzed within one day to determine the intraday variability, and the same sample was used on six independent days to obtain the interday variability. The quantity of each ingredient in the sample was determined from its corresponding calibration curve. The relative standard deviation (RSD) obtained by six replicated injections of the solution was taken as a measure of method repeatability. As shown in Table 2, the intra- and interday RSD values of the three compounds are all less than 2.5%, implying good reproducibility.

The recovery test was done by spiking a solution containing known quantities of the standard and known amounts of powdered mulberry samples, mixed prior to extraction. The standard solutions with their concentration levels in the middle part of the calibration curve and six fortified samples were applied. The recovery rate of the method was 100.0–103.5%, with RSD less than 2.5%, suggesting that the method is accurate (Table 2).

Stability of the solutions of the samples was tested by comparing sample solutions that were kept at room temperature with the standard solutions every 2 h within 24 h, and we found that the sample solutions were stable within 12 h (RSD < 1.7%).

## 3. Results and Discussion

**Figure 2:**
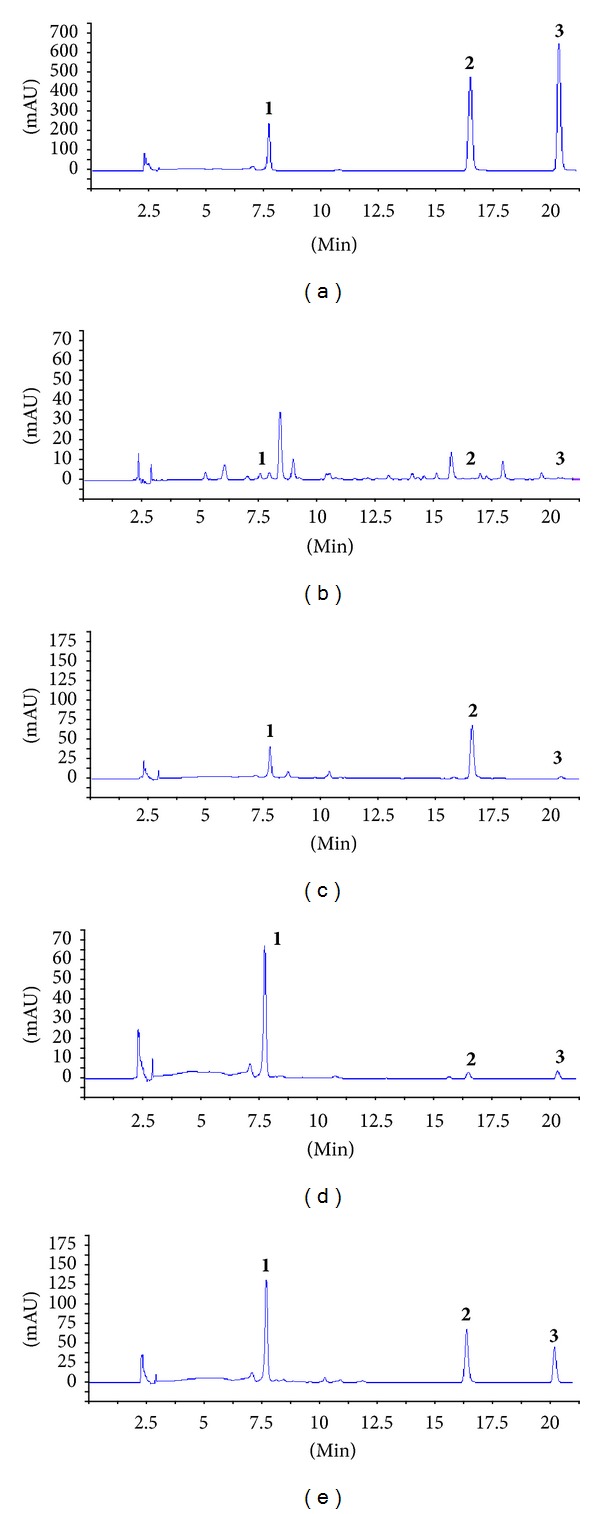
HPLC chromatograms of standard mixture (a), leaves of *Morus atropurpurea* Roxb (b), stem of *Morus atropurpurea* Roxb (c), bark of *Morus atropurpurea* Roxb (d), and roots of *Morus atropurpurea* Roxb (e). **1** mulberroside A; **2** oxyresveratrol; **3** resveratrol.

All three stilbenes in mulberries were evaluated by a developed analytical method as described in what follows. Peaks in the achieved chromatograms were recognized by comparing the retention times and ultraviolet spectra with those of standard solutions. Representative chromatograms are shown in Figure 2. Retention parameters for **1**–**3** were 7.65, 16.33, and 20.15 min, respectively.

The complete summary of the results of mulberroside A, oxyresveratrol, and resveratrol from different portions of *Morus atropurpurea* Roxb, *Morus alba* Linn, and *Morus latifolia* Poir collected in different seasons is shown in Tables 3 and 4 and that from seedlings of *Morus atropurpurea* Roxb is shown in Table 5.

In this study, we found that mulberroside A was richest in bark and roots in September, oxyresveratrol was richest in roots and stem in September as well, and resveratrol was very low in all. Mulberroside A levels were the highest in the bark and roots of *Morus atropurpurea* Roxb, *Morus alba* Linn and *Morus latifolia* Poir. In the stem, *Morus latifolia* Poir contained the highest level of mulberroside A, but it was undetected in the leaves of *Morus atropurpurea* Roxb, *Morus alba* Linn, and *Morus latifolia* Poir.

Although they have been found in mulberry wood [10], the amount and the relative quantity of oxyresveratrol and resveratrol in mulberry bark, pith, roots, or tuber were uncertain. In the current study, we found that many parts of mulberry contained more oxyresveratrol than resveratrol (Tables 3 and 4), and in general, the levels of both are much less than mulberroside A. September is the best time to obtain both oxyresveratrol and resveratrol. It seems that the parts and varieties of mulberry, as well as season, are all factors influencing the levels of mulberroside A, oxyresveratrol, and resveratrol.

Glycosylation of polyphenolic compounds is a common feature in plants, which can enhance the stability of compounds [11]. In the case of glycosylation of stilbenes, it may protect them from oxidation and enzymatic degradation and thus enhances their stability. In the processes of glycosylation of stilbenes, free stilbene is first synthesized and then glycosylated by endogenous glycosyltransferases [12].

The major role of stilbenes in a number of plant families, such as peanut, mulberry, and grapevine, is working as phytoalexins [13], a group of low-weight molecules with protective functions produced by plants in response to infection [14]. In this context, our finding of a higher level of stilbenes in tubers than in other parts of *Morus atropurpurea* Roxb (Table 4) is expected.

**Figure 3:**
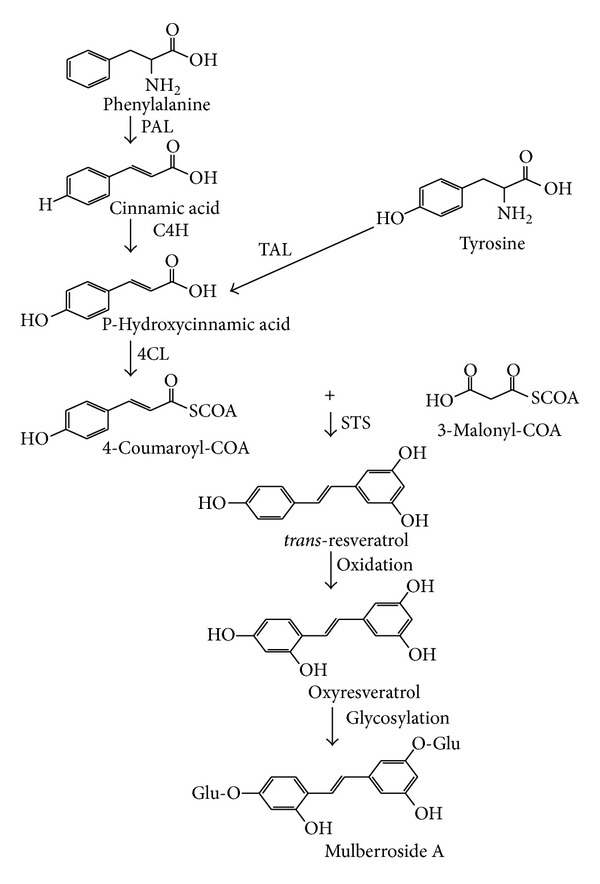
Biosynthesis of resveratrol via the phenylalanine/polymalonate pathway. Phenylalanine ammonia lyase (PAL); tyrosine ammonia lyase (TAL); cinnamate-4-hydroxylase (C4H); 4-coenzyme A ligase (4CL); stilbene synthase (STS). Adapted from Jeandet et al. [12].

The formation of stilbene phytoalexins involves the phenylalanine/polymalonate route (Figure 3), and the key step in this biosynthesis pathway is catalyzed by stilbene synthase (STS), which exerts iterative condensation reactions with malonyl-CoA [12, 15, 16]. With the starter coenzyme A-esters of cinnamic acid derivatives (p-coumaroyl-CoA in the case of resveratrol or cinnamoyl-CoA in the case of pinosylvin) and three malonyl-CoA units, STS can produce the stilbene phytoalexins in one reaction. Leaves which were suggested to be the site of stilbene biosynthesis as two peaks of STS mRNAs in grapevine leaves treated by ultraviolet light were observed by Douillet-Breuil et al. [17]. In the current study, stilbenes were found to be richest in roots, suggesting that there may be another site for stilbene biosynthesis.

In order to get new insight about stilbene biotransformation, we examined three major stilbenes in leaves and roots of seedlings of mulberry. This is the first time, to the best of our knowledge, that the amount of mulberroside A, oxyresveratrol, and resveratrol in *Morus atropurpurea *Roxb seedling leaves and roots has been quantified (Table 5). Among *Morus atropurpurea* Roxb seedlings of the 7th day–20th day, mulberroside A was not detected in the leaves, while it varied between 354.2 and 1128.1 *μ*g g^−1^ in the roots and reached its peaks on the 13th day and in 20th day, respectively. Oxyresveratrol peaked on the 17th day with 114.2 *μ*g g^−1^ in leaves and 193.8 *μ*g g^−1^ in roots. In leaves, on the 9th day, the level of resveratrol was 226.8 *μ*g g^−1^, while oxyresveratrol was only 47.2 *μ*g g^−1^. Resveratrol has two peaks in roots, 220.2 *μ*g g^−1^ on the 7th day and 211.2 *μ*g g^−1^ on the 13th day. As in both leaves and roots, the peaks for resveratrol always run ahead of oxyresveratrol in time, it is logical to propose that oxyresveratrol is probably transformed from resveratrol through oxidation.

## 4. Conclusions

The levels of the stilbenes vary in different parts of varieties of mulberries collected in different seasons and in the seedlings of *Morus atropurpurea* Roxb. The method has been proved to be simple, rapid, and accurate and can be readily used to determine the content of the major stilbenes in mulberries.

## Figures and Tables

**Table 1:** Linear relation between peak area and concentration (*n* = 6).

Compound	Regression equation^a^	*r* ^2^ ^b^	Linear range (*μ*g mL^−1^)	LOD^c^ (ng mL^−1^)	LOQ^d^ (ng mL^−1^)
**1**	*y* = 22.804*x* − 25.360	0.9999	0.64–404.40	2.60	8.67
**2**	*y* = 54.197*x* − 144.350	0.9999	0.71–444.00	2.10	7.00
**3**	*y* = 75.172*x* − 71.439	1	0.72–448.00	0.95	3.17

^a^In the regression equation *y* = *ax* + *b*, *x* refers to the concentration of the compound (*μ*g mL^−1^), *y* the peak area.

^b^ *r* ^2^ is the correlation coefficient of the equation.

^c^LOD: limit of detection.

^d^LOQ: limit of quantification.

**Table 2:** Intra- and interday repeatability and recovery of the three major stilbenes in mulberry.

Compound	Repeatability (*n* = 6)				Recovery (*n* = 6)	
	Intraday Mean ± S.D.^a^	R.S.D. (%)	Interday Mean ± S.D.	R.S.D. (%)	Mean (%)^b^	R.S.D. (%)^c^
**1**	1140.2 ± 12.5	1.1	1138.0 ± 14.8	1.3	103.5	2.4
**2**	840.3 ± 9.2	1.1	846.4 ± 14.4	1.7	102.4	1.9
**3**	61.2 ± 0.9	1.5	60.8 ± 1.4	2.3	100.0	2.3

^a^Data were *μ*g constituents per gram drug.

^b^Calculated as detected amount/added amount × 100%. Data were means of six experiments.

^c^R.S.D. (%) = (S.D./mean) × 100.

**Table 3:** Contents of mulberroside A, oxyresveratrol, and resveratrol in the different portions of *Morus atropurpurea *Roxb,* Morus alba *Linn, and *Morus latifolia *Poir in September (*n* = 3).

Sample	*Morus atropurpurea *Roxb (*μ*g g^−1^)			*Morus alba *Linn (*μ*g g^−1^)			*Morus latifolia* Poir (*μ*g g^−1^)		
	Mulberroside A	Oxyresveratrol	Resveratrol	Mulberroside A	Oxyresveratrol	Resveratrol	Mulberroside A	Oxyresveratrol	Resveratrol
Leaves	nd	nd	nd	nd	144.5 ± 1.6	nd	nd	147.2 ± 1.6	nd
Stem	182.4 ± 2.0	680.6 ± 7.5	38.2 ± 0.6	201.7 ± 2.2	60.7 ± 0.7	37.1 ± 0.6	2010.8 ± 22.1	2153.5 ± 23.7	47.6 ± 0.7
Bark	4278.1 ± 47.1	150.8 ± 1.7	82.6 ± 1.2	2631.6 ± 28.9	138.8 ± 1.5	37.4 ± 0.6	2097.9 ± 23.1	151.1 ± 1.7	51.4 ± 0.8
Roots	17110.1 ± 188.2	634.6 ± 7.0	98.2 ± 1.5	5131.9 ± 56.5	374.6 ± 4.1	63.9 ± 1.0	8766.0 ± 96.4	666.3 ± 7.3	49.1 ± 0.7

nd: not detected.

**Table 4:** Contents of mulberroside A, oxyresveratrol, and resveratrol in the different portions and different seasons of *Morus atropurpurea *Roxb (*n* = 3).

Sample	Mulberroside A (*μ*g g^−1^)				Oxyresveratrol (*μ*g g^−1^)				Resveratrol (*μ*g g^−1^)			
	March	June	September	December	March	June	September	December	March	June	September	December
Leaves	nd	nd	nd	nd	66.4 ± 0.7	45.4 ± 0.5	nd	40.9 ± 0.4	56.3 ± 0.8	54.5 ± 0.8	nd	113.1 ± 1.7
Stem	386.5 ± 4.3	321.4 ± 3.5	182.4 ± 2.0	879.0 ± 9.7	64.1 ± 0.7	548.3 ± 6.0	680.6 ± 7.5	579.9 ± 6.4	41.1 ± 0.6	38.3 ± 0.6	38.2 ± 0.6	41.8 ± 0.6
Bark	3454.9 ± 38.0	4937.1 ± 54.3	4278.1 ± 47.1	2942.6 ± 32.4	53.1 ± 0.6	114.9 ± 1.3	150.8 ± 1.7	47.5 ± 0.5	42.5 ± 0.6	40.7 ± 0.6	82.6 ± 1.2	38.4 ± 0.6
Roots	10550.8 ± 116.1	13728.6 ± 151.0	17110.1 ± 188.2	17011.6 ± 187.1	88.3 ± 1.0	235.5 ± 2.6	634.6 ± 7.0	479.6 ± 5.3	65.9 ± 1.0	39.2 ± 0.6	98.2 ± 1.5	72.5 ± 1.1
Pith	348.1 ± 3.8	407.2 ± 4.5	1050.2 ± 11.6	511.8 ± 5.6	41.0 ± 0.5	840.8 ± 9.2	220.4 ± 2.4	132.3 ± 1.5	52.0 ± 0.8	47.2 ± 0.7	88.6 ± 1.3	39.3 ± 0.6
Tuber	8773.3 ± 96.5	11122.7 ± 122.3	13007.2 ± 143.1	7118.8 ± 78.3	49.2 ± 0.5	77.8 ± 0.9	900.7 ± 9.9	313.1 ± 3.4	92.3 ± 1.4	41.4 ± 0.6	38.8 ± 0.6	41.0 ± 0.6

nd: not detected.

**Table 5:** Contents of components of *Morus atropurpurea *Roxb seedling (*n* = 3).

Sample	Mulberroside A (*μ*g g^−1^)	Oxyresveratrol (*μ*g g^−1^)	Resveratrol (*μ*g g^−1^)
Leaves (7th day)	nd	39.0 ± 0.4	37.5 ± 0.6
Leaves (9th day)	nd	47.2 ± 0.5	226.8 ± 3.4
Leaves (11st day)	nd	51.1 ± 0.6	44.2 ± 0.7
Leaves (13rd day)	nd	69.4 ± 0.8	76.0 ± 1.1
Leaves (15th day)	nd	70.8 ± 0.8	46.2 ± 0.7
Leaves (17th day)	nd	114.2 ± 1.3	91.7 ± 1.4
Leaves (20th day)	nd	67.0 ± 0.7	42.4 ± 0.6
Roots (7th day)	354.2 ± 3.9	51.7 ± 0.6	220.2 ± 3.3
Roots (9th day)	362.4 ± 4.0	69.1 ± 0.8	175.9 ± 2.6
Roots (11st day)	413.8 ± 4.6	71.9 ± 0.8	108.1 ± 1.6
Roots (13rd day)	1128.1 ± 12.4	82.4 ± 0.9	211.2 ± 3.2
Roots (15th day)	923.7 ± 10.2	129.1 ± 1.4	111.4 ± 1.7
Roots (17th day)	631.0 ± 6.9	193.8 ± 2.1	73.1 ± 1.1
Roots (20th day)	1015.2 ± 11.2	55.9 ± 0.6	43.6 ± 0.7

nd: not detected.
